# Orthopedic guidelines: Relevance

**DOI:** 10.4103/0019-5413.80035

**Published:** 2011

**Authors:** Augusto Sarmiento

**Affiliations:** *Department of Orthopaedics, University of Miami School of Medicine, Miami, Florida, USA*

With great enthusiasm and élan the American Academy of Orthopedic Surgeons’ Joint Registry has taken a center stage in America and many have taken for granted its success is beyond questioning.

Despite the fact the concept is laudable and sound, I question the romantic conclusion, first because it has, much too quickly, been built on the belief that the alleged success of similar registries in the Scandinavian countries, can be easily duplicated in other countries. Such a premise fails to recognize the profound differences in the environment into which they have flourished in Europe and the environment in the United States as well as in many other developing countries. The smaller population of the Northern countries, their semi-socialized systems of medical care delivery, their much smaller number of different implants used, and the orthopedists’ disciplined cooperation with national projects will not find similar fertile grounds in other countries. That being the case, it is very likely that when few institutions participate in the effort there will not be appropriate representation of the surgical practices of the larger orthopedic communities. The participating surgeons will likely be members of the same social clubs, often called orthopedic societies, who will bring into the picture the inborn prejudices that we all carry with us, no matter where our destination is proposed to be. With great frequency we see well-intentioned people deeply committed to a particular agenda unintentionally distort facts and compromise progress.

A mechanism to ascertain the veracity of the information provided to the main repository does not exist, as demonstrated by the revelations recently brought forward by the United States’ Justice Department Investigation of what it calls major and rampant ethical transgressions in the relationship between orthopedics and industry.^1^ People of the caliber of Professor Carr, from Oxford University, have, among many others, asked which research is to be believed.^2^

Twenty-five years ago, while serving as chair of the Committee on Injuries of the American Academy of Orthopedic Surgeons (AAOS), I proposed and received approval for the establishment of a National Fracture Registry. One year later I asked the Board of Directors to cancel the project, not because the computer sophistication was not well developed, but because I quickly discovered that the data being reported to the central office had been too often fabricated or manipulated. It was human frailty that killed the dream.

Although the day will come when the many hurdles confronting registries will be overcome, such a day has not as arrived. Additional work on the project should continue uninterruptedly in order to expedite the birth of a viable structure.^3^ Casual assumptions should be held up to relentless scrutiny, and Panglossian optimism should be tempered. Otherwise, the possibility exists that 10 years from now we will have nothing more than millions pieces of data but no tangible information from which knowledge, much less wisdom, can be extracted. The experience of Professor Maurice Muller in Switzerland with his tireless four-decade work on a Documentation Center should be kept uppermost in our minds. Millions of dollars were spent into the effort, which, however, never brought about concrete benefits.

I address today Orthopedic Guidelines, a relatively new concept, which as in the case of registries has brought forth a great deal of enthusiasm and support at high levels of the American orthopedic apparatus. How much of the enthusiasm is infatuation with the new system, mistakenly assuming it is better than the old, is yet to be known. This new kid on the block has already generated controversies, and I suspect as a result of insufficient preparation before releasing them to the community. I have thus far concluded that guidelines for every orthopedic condition will not accomplish what the Academy anticipates; in contrast, they could become a problem to the Academy and to its fellowship. They are not supposed to be mandates, but over time they could easily become dicta, with which many will feel comfortable, since the need for independent thinking is not necessary any longer. I have familiarized myself with the guidelines for fractures of the distal radius, but not with other more recently released.^4^ While studying those guidelines I learned that the authors of the guidelines had allegedly reviewed over 1000 articles. From these, 96 were included in their final report while approximately 867 were excluded as not meeting the necessary criteria, such as evidence-based confirmation, whatever that means. However, a casual glance at the “included” articles showed that at least 30 were unrelated to the subject of wrist fractures and dealt with general views on statistical methods, while others were simply descriptions of techniques in fracture management or observations of correlations between fractures of the spine or hip with wrist fractures. Two articles dealt with the use of Vitamin C in the care of fractures and both were written by the same author.

The authors state, “The following recommendations have adequate evidence to support a moderately strong endorsement. (italics added)…. We suggest operative fixation as opposed to cast fixation for fractures with postreduction radial shortening greater than 3 mm, dorsal tilt greater than 10°, or intraarticular displacement or step-off greater than 2 mm…. We suggest adjuvant treatment of distal radial fractures with Vitamin C for the prevention of disproportional pain.”^4^

Rather conspicuous from their absence were articles recently published in peer-reviewed journals discussing longer-term follow-up. One such publication in *The Journal of Bone & Joint Surgery (Am.)* indicated that at the end of one year the “minor limitations in the range of motion and diminished grip strength seen in the nonoperative care do not seem to limit functional recovery.”^5^ Although claims are made regarding the lack of conflict of interest among the authors of the guideline, most of them acknowledged association with industry, which might have, consciously or unconsciously, affected their views.

The guidelines do not say that anatomical/radiographic deviations or failure to prescribe Vitamin C are synonymous with malpractice, but some patients, legitimately or with self-seving ulterior motives, will claim to be unhappy with their results; and having learned *via* direct-consumer marketing what the American Academy of Orthopedic Surgeons’ guidelines say, will obtain the services of attorneys, who will prosecute surgeons who departed from the “wisdom” of the guidelines. The guidelines of AAOS have a wider acceptability. The use of such guidelines in a developing countries disregarding infrastructure, quality of instrumentation and implants and level of surgical training will produce lots of surgical disasters before it is realized.^6^

We all know there are circumstances dictated by a variety of reasons, such as patients’ age, underlying diseases, or many others, when greater degrees of radiologically measured deviations from the normal are clinically inconsequential. Even otherwise the outcome of a particular treatment modality will vary with viariation in infrastructure, disease state and the patient.

To accept without questioning the judiciousness and smartness of a small group of experts may be wrong, for after all they are humans carrying with them the weaknesses we all possess. Plato, in the description of the ideal republic, suggested that a philosopher/king should be leader of the nation, who in turn would be assisted by philosophers/guardians. A few centuries later, Juvenal, the famed Roman poet, discussing Plato’s ideas asked, “Who will guard the guardians?”

Wait till guidelines appear regarding fractures of the clavicle if they have not already been released. I anticipate that an epidemic of surgery will occur and that the surgical approach will be applied to virtually all such factures. Any shortening, displacement, location, or comminution may be reasons to “highly recommend” the surgical intervention. To support my suspicion, a recently published article can be used. It compared the results from surgical versus the nonsurgical treatment of displaced clavicular fractures. The following conclusions were drawn: The mean time to radiographic union was 28.4 weeks in the nonoperative group compared with 16.4 weeks in the operative group (*P* = 0.001). There were two nonunions in the operative group compared with seven in the nonoperative group (*P* = 0.042). Symptomatic malunion developed in nine patients in the nonoperative group and in none in the operative group (*P* = 0.001). At one year after the injury, the patients in the operative group were more likely to be satisfied with the appearance of the shoulder (*P* = 0.001) and with the shoulder in general (*P* = 0.002) than were those in the nonoperative group.^7^ These reported results run contrary to long-standing orthopedic practices, that for many a generation had, without equivocation, recognized the excellent results in the overwhelming majority of these fractures managed nonsurgically.

Nothing is said in any publications recommending the surgical approach about the fact that the shortening and degree of displacement seeing on radiographs can be made to appear greater or lesser according to the direction of the X-ray beam.

I suspect that some orthopedists will embrace the new dogma, either from fear of being accused of ignoring the gospel, while other will perform surgery, whether needed or not, in order to reap the additional financial benefits that surgery brings. This will inevitably increase the already exponentially growing cost of orthopedic care and will add some percent of operative complications in the hands of these with not an ideal infrastructure.

Final conclusions cannot be drawn at this time as to the wisdom being displayed in pursuing the several projects I have addressed. I cannot help but suspect that the probability exists that many of them, having been nothing more than dreams, will fade from the scene in a short time after suffering the inexorable fate of dreams.

Our profession is facing major challenges begging for resolution. Wasting time, effort, and money addressing inconsequential issues are not answers. Our representative organizations and we, the practitioners of the art, must concentrate on fundamental ones, such as the growing loss of professionalism in our ranks, the embarrassing control of education by the pharmaceutical and the implant manufacturing companies; the exaggerated commercialization of orthopedics; the erosion of its territory by other medical and allied health professions; the inadequately addressed potential crisis created by an exaggerated emphasis on fellowships for every graduating resident, now being aggravated by the practice of having fellowships subsidized by surgical implant companies; the soon to become evident shortage of orthopedists particularly in smaller communities.

We are not children in need of clearly established norms of conduct. Neither the Academy nor other organization has the authority to recommend to us which are the treatments highly recommended or not recommended at all. Their role is to serve as effective vehicles for the dissemination of knowledge; knowledge that we currently acquire from hundreds of journals and books, thousands of scientific meetings where presentations for and against a variety of treatment modalities are debated. The orthopedists have always used and should continue to use that information to make their own judgment in determining what in their opinion is the rational treatment modality according to specific circumstances.

We do not, at this time, need Orthopedic Guidelines of the proposed format for every condition.^8^ Any system, except in very serious circumstances, that interferes with independent thinking and constrains critical inquiry must be avoided. History is replete with examples where governments or religious organizations considered themselves powerful enough to impose regulations that had to be followed to the letter by all citizens, resulting in catastrophic consequences. Industry’s illegal and unprofessional actions to silence the voices of those who express opinions thought to affect its financial profits must be brought to an end as soon as possible before is too late.^9^ Peer-reviewed orthopedic journals should temper exaggerated profit-driven emphasis and in doing so risking quality.

Major changes are taking place around the world concerning healthcare delivery. Change cannot be avoided and will, sooner or later, arrive. If we do not have the courage to bring the necessary changes from above, it will come from the bottom, which then will be associated with undesirable consequences, because the change took place under an atmosphere guided by emotion and rhetoric rather than one where reason prevails. If our passivity continues unharnessed, by the time we are proven wrong the damage already had already been done.
